# Habitat shifts in the evolutionary history of a Neotropical flycatcher lineage from forest and open landscapes

**DOI:** 10.1186/1471-2148-8-193

**Published:** 2008-07-07

**Authors:** Frank E Rheindt, Les Christidis, Janette A Norman

**Affiliations:** 1 Department of Genetics, University of Melbourne, Parkville Campus, Melbourne VIC 3000, Australia; 2 Population Ecology and Genetics Unit, Sciences Department, Museum Victoria, 11 Nicholson St., Carlton VIC 3053, Australia; 3 Australian Museum, 6 College Street, Sydney NSW 2010, Australia

## Abstract

**Background:**

Little is known about the role ecological shifts play in the evolution of Neotropical radiations that have colonized a variety of environments. We here examine habitat shifts in the evolutionary history of *Elaenia *flycatchers, a Neotropical bird lineage that lives in a range of forest and open habitats. We evaluate phylogenetic relationships within the genus based on mitochondrial and nuclear DNA sequence data, and then employ parsimony-based and Bayesian methods to reconstruct preferences for a number of habitat types and migratory behaviour throughout the evolutionary history of the genus. Using a molecular clock approach, we date the most important habitat shifts.

**Results:**

Our analyses resolve phylogenetic relationships among *Elaenia *species and confirm several species associations predicted by morphology while furnishing support for other taxon placements that are in conflict with traditional classification, such as the elevation of various *Elaenia *taxa to species level. While savannah specialism is restricted to one basal clade within the genus, montane forest was invaded from open habitat only on a limited number of occasions. Riparian growth may have been favoured early on in the evolution of the main *Elaenia *clade and subsequently been deserted on several occasions. Austral long-distance migratory behaviour evolved on several occasions.

**Conclusion:**

Ancestral reconstructions of habitat preferences reveal pronounced differences not only in the timing of the emergence of certain habitat preferences, but also in the frequency of habitat shifts. The early origin of savannah specialism in *Elaenia *highlights the importance of this habitat in Neotropical Pliocene and late Miocene biogeography. While forest in old mountain ranges such as the Tepuis and the Brazilian Shield was colonized early on, the most important colonization event of montane forest was in conjunction with Pliocene Andean uplift. Riparian habitats may have played an important role in facilitating habitat shifts by birds expanding up the mountains along streams and adapting to newly emerging montane forest habitat.

## Background

The major evolutionary mechanisms that have resulted in the Neotropical Region's extremely large and diverse fauna are not well understood. Most phylogenetic studies have concentrated on radiations that have diversified within Neotropical forests [1-9] and a few have dealt with radiations centered in open habitats such as grasslands and open scrub [10-13]. These studies have found that the most recent speciation events have occurred between geographically isolated populations inhabiting the same habitat type, suggesting that allopatric isolation through vicariance has been a dominant speciation mechanism [1,3,6,9]. A limitation in extrapolating from these studies is that most research has focussed on lineages that occur in different microhabitats within one single habitat type (e.g. *terra firme *forest, floodplain forest, montane forest). Additionally, suitable methods for the reconstruction of habitat preferences of ancestral lineages have been limited until recently [14]. What is lacking is data on radiations that include opposite ends of a spectrum of habitats, such as dense forest and open savannah. Such studies will provide insights into the evolutionary significance of habitat shifts following colonization of new habitats.

**Figure 1:**
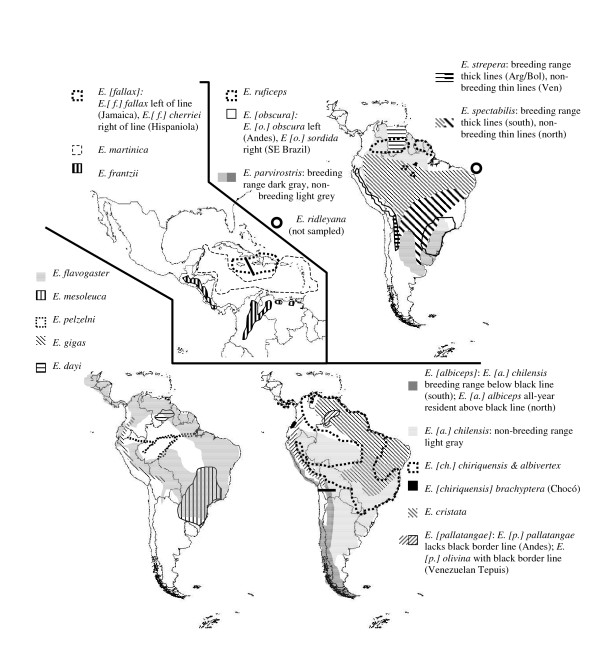
Distribution maps for all species-level lineages in the genus *Elaenia*.

We investigated the evolutionary history of Neotropical *Elaenia *flycatchers (Aves; Tyrannidae) using both mitochondrial and nuclear DNA sequences. This genus is distributed over a range of forest and open habitats from Mexico and Hispaniola to the southern tip of South America [15,16] (Figure 1; Table 1). While largely avoiding tropical lowland rainforest during the breeding season, the genus is represented in a variety of other dense forest habitats. In addition, the genus is widespread in scrubby and open tree habitats in both the Neotropical highlands and lowlands (Table 1). Austral long-distance migration is well represented in the genus [15,16].

**Table 1:** Wing-bar count and some ecological parameters for all species-level lineages of *Elaenia *sampled

Taxon	Wing-bars	Movements	Appr. preferred breeding elevation [m]	Preferred spectrum of breeding habitat
*E. martinica*	2	sedentary	0 – 1000	Scrub to forest
*E. flavogaster*	2	sedentary; some Mexican populations migrate	0 – 1500	Scrub/savannah to light woodland, also riparian growth
*E. spectabilis*	3	austral migrant	0 – 800	Forest border and thickets, riparian growth (heavier vegetation than *E. flavogaster*)
*E. albiceps*	2	austral migrant (*E. a. chilensis*) and sedentary	0 – 3300	Scrub to beech forest
*E. parvirostris*	3	austral migrant	0 – 1000	Forest border to open areas, riparian vegetation
*E. mesoleuca*	2	mostly sedentary, southern populations migratory	0 – 2000	Forest, gallery forest
*E. strepera*	2	austral migrant	500 – 2000	Forest border, streamside vegetation
*E. gigas*	2	sedentary	350 – 1500	Riparian growth, clearings
*E. pelzelni*	3	sedentary	0 – 200	Riparian growth along Amazon, river-island habitat
*E. cristata*	2	mostly sedentary, some populations migratory	0 – 1500	Savannah and cerrado
*E. [chiriquensis] albivertex*	2	mostly sedentary, some populations migratory	0 – 2000	Scrub, cerrado, open woodland, riverine vegetation
*E. [chiriquensis] brachyptera*	2	sedentary	700 – 2800	Forest border to lighter woodland
*E. ruficeps*	2	sedentary	0 – 1400	Cerrado, savanna, white-sand formations
*E. frantzii*	2	sedentary, some altitudinal and latitudinal migration	750 – 3600	Forest to borders
*E. [f.] fallax*	2	sedentary	500 – 2000	Forest to borders
*E. [f.] cherriei*	2	sedentary	500 – 2000	Forest to borders
*E. [o.] obscura*	2	sedentary	1700 – 3000	Forest to woodland
*E. [o.] sordida*	2	sedentary	0 – 2000	Forest to woodland
*E. dayi*	2	sedentary	1800 – 2600	Forest to stunted savanna
*E. [p.] pallatangae*	2	sedentary	1500 – 3000	Forest border to scrub
*E. [p.] olivina*	2	sedentary	900 – 2400	Forest border to scrub

Data from references [15,16]

Our study included samples of all but one currently recognised species (Table 2) and focused on the reconstruction of ancestral habitat preferences using both parsimony and Bayesian approaches. We traced the preferences for certain habitat types throughout the evolutionary history of the genus, namely forest, savannah, and riparian habitat, and estimated relative rates of transition between the occupancy of those habitats. Additionally, we reconstructed migratory behavior of the ancestors of *Elaenia *to trace the origin of austral long-distance migration in the genus and assess its role in the diversification of the genus. These data, in addition to molecular clock dating of speciation events, were then used to derive conclusions about modes of speciation and the evolution of habitat preference in *Elaenia*.

**Table 2:** Genbank accession numbers, genetic tissue numbers, institutions and collection localities of molecular specimens

Genbank Accession Number: (ND2, Fib5) or (ND2 only)	Species	Tissue Number	Institution	Locality
EU311054, EU311115	*Elaenia albiceps*	430029	FMNH	PERU: Cusco
EU311053, EU311136	*Elaenia albiceps*	PRS 1734	AMNH	ARGENTINA: Neuquén, Anelo, Sierra Auca Mahuida
EU310951, EU310939	*Elaenia [chiriquensis] brachyptera*	Rheindt et al.: Cryptic speciation in the Lesser Elaenia *Elaenia chiriquensis*, submitted		ECUADOR: Esmeraldas, 10 km W Lita, c. 1000 m
EU310947, EU310940	*Elaenia [chiriquensis] albivertex*			BRAZIL: Amapa
EU310948, EU310941	*Elaenia [chiriquensis] albivertex*			BOLIVIA: Sta Cruz, Velasco, Pre Parque Nacional Noel Kempff Mercado, 30 km E Aserradero Moira
DQ294543, DQ294455	*Elaenia [chiriquensis] albivertex*	Tello and Bates [43]		BRAZIL: Amapa, Tartarugalzinho, Lago Cujubim
EU311090, EU311125	*Elaenia cristata*	392566	FMNH	BRAZIL: Para
EU311055	*Elaenia cristata*	B11919	SNMNH	GUYANA
EU311063, EU311132	*Elaenia cristata*	B11991	SNMNH	GUYANA
EU311067	*Elaenia cristata*	B14636	LSU	BOLIVIA: Sta Cruz, Serrania de Huanchaca, 21 km SE Catarata Arco Iris
EU311094, EU311123	*Elaenia dayi*	GFB 2837	AMNH	VENEZUELA: Bolivar, Auyan Tepui, Camp V
EU311052, EU311122	*Elaenia fallax*	331075	FMNH	JAMAICA
EU311050, EU311113	*Elaenia fallax*	JAG 2175	AMNH	DOMINICAN REP.: Independencia, Sierra de Neiba
EU311089, EU311145	*Elaenia fallax*	KU 6293	KU	DOMINICAN REPUBLIC
EU311070	*Elaenia flavogaster*	391465	FMNH	BRAZIL: Amapa
EU311082, EU311116	*Elaenia flavogaster*	393044	FMNH	COSTA RICA
EU311047	*Elaenia flavogaster*	394494	FMNH	TRINIDAD & TOBAGO: Tobago
EU311093	*Elaenia flavogaster*	B1810	SNMNH	PANAMA
EU311091, EU311118	*Elaenia flavogaster*	B2112	SNMNH	St. VINCENT
EU311060, EU311117	*Elaenia flavogaster*	B4362	SNMNH	GUYANA
EF501906, EF501840	*Elaenia flavogaster*	Rheindt et al. [17]		BOLIVIA: Sta Cruz, Serrania de Huanchaca, 21 km SE Catarata Arco Iris
EU311049, EU311120	*Elaenia frantzii*	B5469	SNMNH	PANAMA
EU311059, EU311143	*Elaenia frantzii*	KU 4901	KU	EL SALVADOR
EU311092, EU311126	*Elaenia gigas*	322868	FMNH	PERU
EU311100, EU311127	*Elaenia gigas*	B22898	LSU	BOLIVIA: La Paz, Prov. B. Saavedra, 68 km by road E Charazani, Quita Calzon
EU311088, EU311124	*Elaenia martinica*	B2116	SNMNH	St. VINCENT
EU311079, EU311119	*Elaenia martinica*	NKK 784	AMNH	CAYMAN ISL.: Grand Cayman, Queens Highway
EU311057, EU311134	*Elaenia martinica*	B11342	LSU	PUERTO RICO: Cabo Rojo, Llanos Costa, 0.5 km NNW mouth Arroyo Cazul
EU311061, EU311141	*Elaenia martinica*	B11343	LSU	PUERTO RICO: Cabo Rojo, Llanos Costa, 0.5 km NNW mouth Arroyo Cazul
EU311077	*Elaenia obscura*	B106786	LSU	BOLIVIA: Beni, Serrania Pilon, 1025 m
EU311083, EU311142	*Elaenia obscura*	B8077	LSU	PERU: Pasco, Playa Pampa, 8 km NW Cushi on trail to Chaglla
EU311087, EU311139	*Elaenia obscura*	B38323	LSU	BOLIVIA: Sta Cruz, La Pajcha ca 28 km S Samaipata
EU311096, EU311144	*Elaenia pallatangae*	GFB 2904	AMNH	VENEZUELA: Bolivar, Cerro Guanay, Camp III
EU311095, EU311146	*Elaenia pallatangae*	GFB 2960	AMNH	VENEZUELA: Amazonas, Cerro Yutaje, 1700 m
EU311048, EU311147	*Elaenia pallatangae*	B8155	LSU	PERU: Pasco, Playa Pampa, 8 km NW Cushi on trail to Chaglla
EU311101, EU311148	*Elaenia pallatangae*	B31835	LSU	PERU: Cajamarca, Quebrada Lanchal, 8 km ESE Sallique
EU311081, EU311114	*Elaenia parvirostris*	334473	FMNH	BOLIVIA: El Beni
EU311075	*Elaenia parvirostris*	ALP 142	AMNH	BOLIVIA: Santa Cruz, Comunidad Karapari, Estancia San Julian, 1000 m W of Rio Parapeti
EU311084	*Elaenia parvirostris*	B5910	SNMNH	ARGENTINA
EU311073	*Elaenia parvirostris*	JJW 278	AMNH	BOLIVIA: Santa Cruz, Comunidad Karapari, Estancia San Julian, 1000 m W of Rio Parapeti
EU311062	*Elaenia parvirostris*	KU 3417	KU	PARAGUAY
EU311068	*Elaenia parvirostris*	PRS 1099	AMNH	ARGENTINA: Buenos Aires, Partido Escobar
EU311076	*Elaenia parvirostris*	B7268	LSU	PERU: Loreto, Amazonas Isla Pasto, 80 km NE Iquitos, 80 m
EU311064	*Elaenia parvirostris*	ANSP 1405	ANSP	ECUADOR: Santiago, 400 m
EU311072	*Elaenia parvirostris*	ANSP 10253	ANSP	URUGUAY: 17 km N Ruta 20 KM41, Rio Negro
EU311071	*Elaenia parvirostris*	ANSP 10258	ANSP	URUGUAY: Maldonado, ca 3 km NE Pan de Azucar
EU311074, EU311140	*Elaenia parvirostris*	ANSP 10272	ANSP	URUGUAY: Canelones, El Pinar
EU311080, EU311130	*Elaenia pelzelni*	B7320	LSU	PERU: Loreto, Amazonas Isla Pasto, 80 km NE Iquitos, 80 m
EU311086, EU311129	*Elaenia pelzelni*	B7249	LSU	PERU: Loreto, Amazonas Isla Pasto, 80 km NE Iquitos, 80 m
EF501917, EF501829	*Elaenia ruficeps*	Rheindt et al. [17]		BRAZIL: Roraima
EF501904, EF501828	*Elaenia ruficeps*			GUYANA
EU311056	*Elaenia ruficeps*	B11371	SNMNH	GUYANA
EU311069	*Elaenia ruficeps*	PEP 2001	AMNH	VENEZUELA: Amazonas, Unturan
EU311051, EU311121	*Elaenia spectabilis*	399286	FMNH	BRAZIL: Alagoas
EU311078, EU311133	*Elaenia spectabilis*	ALP 150	AMNH	BOLIVIA: Santa Cruz, Comunidad Karapari, Estancia San Julian, 1000 m W of Rio Parapeti
EU311058	*Elaenia spectabilis*	B5975	SNMNH	ARGENTINA
EU311085, EU311131	*Elaenia spectabilis*	KU 3299	KU	PARAGUAY
EU311107	*Elaenia spectabilis*	B42595	LSU	PERU: Loreto, 7 km SW Jeberos
EU311108	*Elaenia spectabilis*	B9559	LSU	BOLIVIA: Pando, Nicolas Suarez, 12 km by road S of Cobija, 8 km W on road to Mucden
EU311103	*Elaenia flavogaster*	125619	ZK	BRAZIL: Para, Belem
EU311105, EU311128	*Elaenia obscura*	125615	ZK	BRAZIL: Andarai, Fazenda Mocambo
EU311110, EU311135	*Elaenia obscura*	127034	ZK	BOLIVIA: Cochabamba, Tablas Montes
EU311102	*Elaenia parvirostris*	126252	ZK	BOLIVIA: Chuquisaca, Sopachuy
EU311104, EU311138	*Elaenia strepera*	126255	ZK	BOLIVIA: Chuquisaca, Sopachuy
EU311106	*Elaenia strepera*	126259	ZK	BOLIVIA: Chuquisaca, Sopachuy
EU311097	*Elaenia strepera*	126249	ZK	BOLIVIA: Chuquisaca, Sopachuy
EU311099, EU311137	*Elaenia strepera*	126251	ZK	BOLIVIA: Chuquisaca, Sopachuy
EU311098	*Elaenia strepera*	126244	ZK	BOLIVIA: Palmarcito
EU311109	*Elaenia strepera*	126245	ZK	BOLIVIA: Palmarcito
EU311065, EU311111	*E. spectabilis*	P2400	LGEMA	BRAZIL: Piaui, P. N. Serra das Confusões
EU311066, EU311112	*E. spectabilis*	P2438	LGEMA	BRAZIL: Piaui, P.N. Serra das Confusões
EU310945, EU310937	*E. mesoleuca*	Rheindt et al.: Cryptic speciation in the Lesser Elaenia *Elaenia chiriquensis*, submitted		BRAZIL: São Paulo, Bananal
EU310944, EU310938	*E. mesoleuca*			BRAZIL: São Paulo, Bananal

Literature references rather than genetic tissue numbers and institutions are given for samples from other studies; abbreviations: FMNH – Field Museum of Natural History, Chicago; AMNH – American Museum of Natural History, New York; SNMNH – Smithsonian National Museum of Natural History, Washington, D.C.; LSU – Louisiana State University Museum of Natural History, Baton Rouge, Louisiana; KU – Kansas University Museum of Natural History, Lawrence, Kansas; ANSP – Academy of Natural Sciences in Philadelphia; ZK – Zoological Museum of the University of Copenhagen; LGEMA – Laboratório de Genética e Evolução Molecular de Aves, São Paulo.

## Results

### Genetic characterization

The aligned Fib5 sequences were 586 bp, 126 bp of which were variable among our samples and 86 bp of which emerged as parsimony-informative. We detected one 5-bp deletion in the sequence of *Capsiempis flaveola *with respect to the in-group as well as eight indels within the in-group, five of which were parsimony-informative. Four parsimony-informative in-group indels could readily be mapped onto our concatenated tree (see below) as a single gain. The remaining indel constituted a 1-bp deletion in both samples of *E. gigas*, the sole sample of *E. [obscura] sordida *and the Bolivian sample of *E. flavogaster*, and required three independent gains. However, the phylogenetic utility of 1-bp deletions has previously been shown to be low in tyrannids [17].

The ND2 partition comprised 1088 bp (incl. up to 47 bp from the flanking tRNA-Met region), with 493 bp being variable and 436 bp parsimony-informative. No anomalies were detected in the translations of the ND2 coding gene, suggesting that the ND2 sequences were of mitochondrial origin.

Chi-square tests of homogeneity of base frequencies across taxa did not show any evidence for base compositional bias (data not shown). Pairwise uncorrected 'p' divergences were calculated for both partitions and those relevant for discussion are presented in Table 3. Saturation graphs with pairwise divergences plotted against the number of transitions/transversions for Fib5 and for all three codons of ND2 (not shown) did not reveal saturation.

**Table 3:** uncorrected 'p' divergences of the ND2 partition for inter- and intra-taxon comparisons

comparison	ND2 uncorrected 'p' divergence [%]
*E. spectabilis ↔ E. pelzelni*	3.5 – 4.4
*E. [f.] fallax ↔ E. [f.] cherriei*	5.1 – 5.3
within *E. [f.] cherriei*	0.1
*E. martinica caymanensis *(Cayman Is.) ↔ other *E. martinica*	1.4
*E. martinica riisii *(Puerto Rico) ↔ *E. m. martinica *(St. Vincent)	0.2
*E. martinica riisii *within Puerto Rico	0
*E. dayi ↔ E. [o.] obscura*	3.8 – 4.2
*E. dayi ↔ E. [obscura] sordida*	4.6
*E. [o.] obscura ↔ E. [o.] sordida*	3.0 – 3.1
within *E. [o.] obscura*	0.1 – 0.3
*E. [p.] pallatangae ↔ E. [p.] olivina*	7.1 – 7.3
*E. albiceps ↔ E. [p.] pallatangae*	0.6
*E. albiceps ↔ E. [p.] olivina*	6.9 – 7.0
within *E. albiceps*	0.4
within *E. [p.] olivina*	0.2
within *E. [p.] pallatangae*	0.2

### Phylogenetic relationships

**Figure 2:**
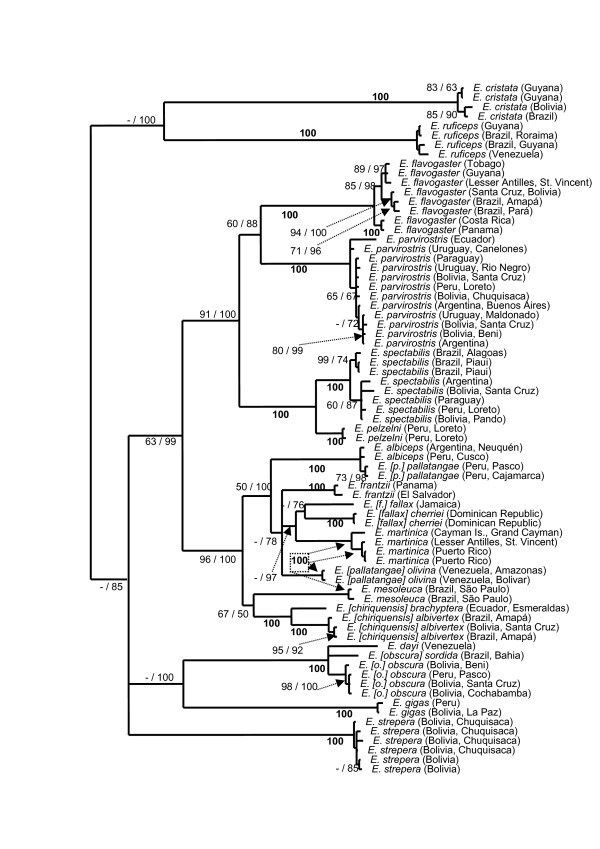
**Bayesian tree of the ND2 partition; numbers at nodes indicate parsimony bootstrap (BS; left) and Bayesian posterior probability (PP; multiplied by 100; right) values;** bold numbers indicate equal support by both types of analysis; only values of BS > 60 and PP > 60 are shown.

BI searches of the ND2 partition yielded the tree depicted in Figure 2. The MP analysis arrived at a consensus topology that was fully congruent with the BI tree (parameters listed in Table 4), although MP bootstrap support was often lower than BI posterior probabilities (Figure 2).

**Table 4:** Maximum-parsimony tree and evolutionary model parameters, Bayesian burn-in specifications for both individual data partitions and for the combined dataset

Partition	ND2	Fib5	Concatenated
Score of MP trees	1578	174	1610
Consistency index of MP trees	0.407	0.759	0.475
Retention index of MP trees	0.844	0.883	0.779
Best evolutionary model (-lnL)	TrN+G (7887.1631)	HKY+G (1937.5226	n/a
Base frequencies (A, C, G)	0.3089, 0.3089, 0.0922	0.2902, 0.1695, 0.2296	n/a
Transition/transversion ratio	n/a	2.4813	n/a
Substitution rate matrix (AC, AG, AT, CG, CT)	1, 29.4411, 1, 1, 15.1094	n/a	n/a
Gamma shape parameter	0.2632	0.3180	n/a
Proportion of invariable sites	0	0	n/a
Burn-in of Bayesian analysis	100,000	10,000	75,000

Abbreviations: MP – most-parsimonious, n/a – not applicable

**Figure 3:**
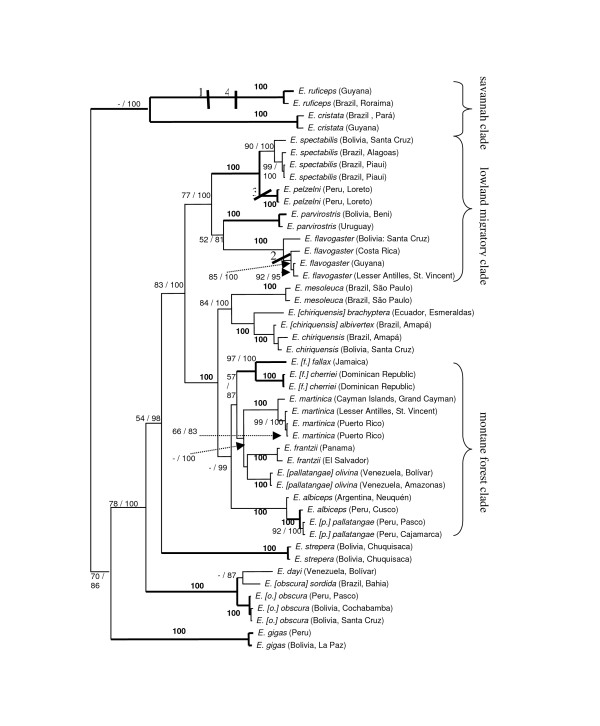
**Bayesian tree of the concatenated dataset;** numbers at nodes indicate parsimony bootstrap (BS; left) and Bayesian posterior probability (PP; multiplied by 100; right) values; bold numbers indicate equal support by both types of analysis; only values of BS > 50 and PP > 80 are shown; thick branches additionally received high support in Fib5 analyses (BS > 80; PP = 100, except for the node uniting all three *E. [o.] obscura*, which only received a PP = 96); numbered cross-bars at nodes refer to parsimony-informative Fib5 indels mapped onto tree: 1.) 10-bp insertion, 2.) 1-bp deletion, 3.) 1-bp insertion, 4.) 1-bp deletion.

Only a limited number of nodes received high support in the MP and BI analyses of the Fib5 partition (tree parameters listed in Table 4). In all but one case, nodes supported by MP bootstrap (BS) values >80 were also supported by BI posterior probabilities (PP) of 100 (Figure 3). None of the nodes supported by Fib5 was in conflict with nodes in the ND2 tree (Figure 2).

In view of the low level of topological conflict between ND2 and Fib5 partitions, we opted to concatenate both datasets. MP and BI searches of the concatenated dataset yielded a well-resolved tree (Figure 3) that displayed almost no conflict to any of the nodes recovered in the ND2 and Fib5 analyses. The only exception involves the position of *E. gigas*, which is recovered as the sister group of the *E. obscura/dayi *super-species by BI analysis of the ND2 partition (Figure 2). However, neither MP analysis of that same partition (Figure 2), nor any type of analysis of the Fib5 partition (Figure 3) identified such a placement. Instead, concatenation of both partitions recovered *E. gigas *as basal to most other *Elaenia *species including the *E. obscura/dayi *super-species (Figure 3). It has previously been shown in tyrant-flycatchers that single markers routinely contain hidden phylogenetic signal that only unfolds once additional markers are added [17], and in view of the congruence of branch support between MP and BI analyses of the concatenated dataset, we suggest that the position of *E. gigas *as depicted in Figure 3 reflects the most likely hypothesis based on our data. As a consequence of the strong topological agreement between the concatenated dataset and single partitions, phylogenetic discussion will be based on the tree resulting from the combined dataset (Figure 3).

### Ecological parameters and timing

**Figure 4:**
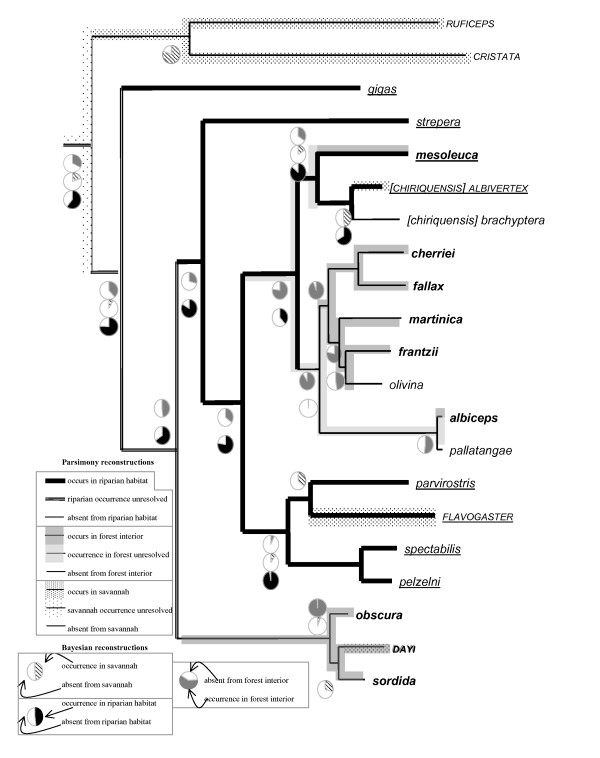
**Occurrence in forest interior, natural savannah/cerrado and riparian habitats mapped onto the *Elaenia *tree topology derived from concatenated dataset with species-level lineages reduced to one representative;** species occurring in **forest interior **are printed bold; species occurring in SAVANNAH are capitalized; species occurring in riparian habitats are underlined; line thickness and line background color refer to parsimony reconstructions; pie charts (only shown for nodes relevant for discussion) refer to Bayesian reconstructions.

**Figure 5:**
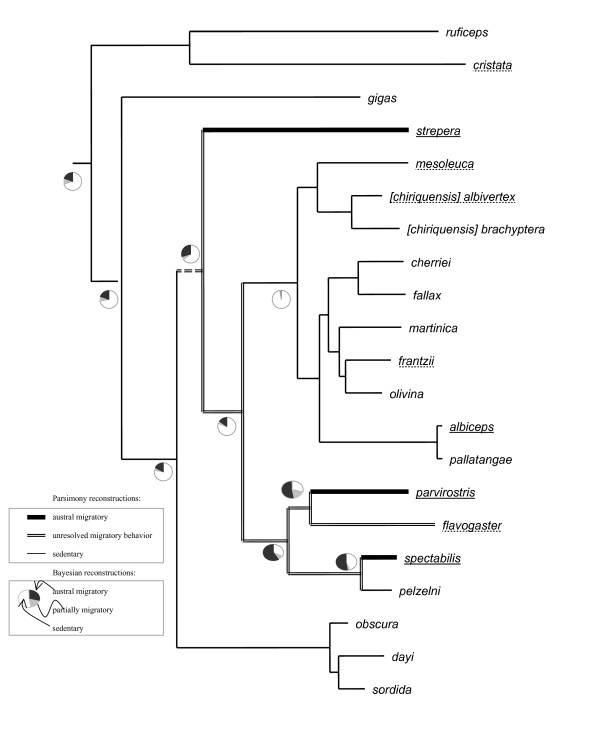
**Migratory behavior mapped onto the *Elaenia *tree topology derived from concatenated dataset with species-level lineages reduced to one representative;**austral migrant species are underlined with a solid line, partially migratory species are underlined with a broken line; sedentary species are not underlined; line thickness refers to parsimony reconstructions; pie charts (only shown for nodes relevant for discussion) refer to Bayesian reconstructions.

MP and BI reconstructions of ancestral migratory behavior and preferences for habitat types are presented in Figure 4 and Figure 5 and are explained more thoroughly in the Discussion. Transition rates between individual parameter states were not significant for any of the characters examined (not shown).

**Figure 6:**
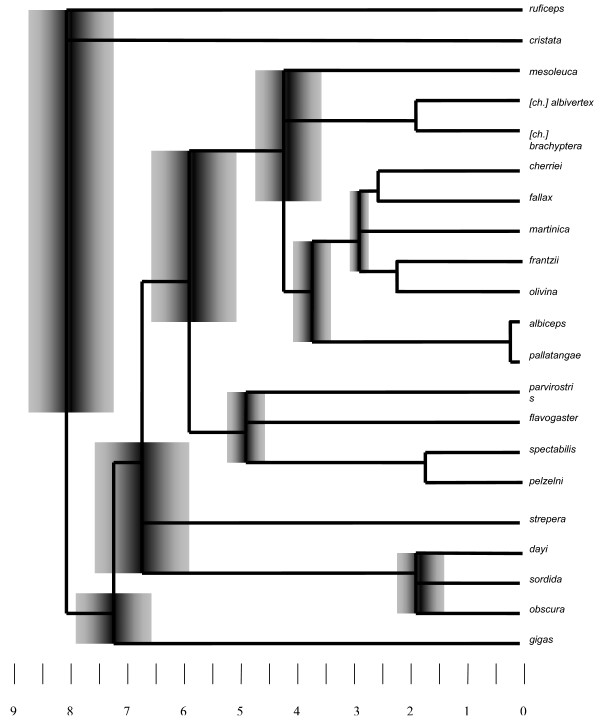
**Age estimates of *Elaenia *speciation events mapped onto the concatenated tree topology using a 2%/million years (MY) molecular clock rate;** scale in MY; error bars at nodes refer to divergence range between taxa; neighboring nodes were merged in cases where both upper and lower divergence bound of one node falls inside the range of the neighbour; scale at bottom indicates millions of years before present.

The likelihood score of the most likely tree computed under relaxed branch-length assumptions was not significantly different from that computed under conditions that enforce a molecular clock (χ^2 ^= 21.56, p = 0.36), which suggests that a molecular clock is a reasonable assumption for the evolution of *Elaenia *flycatchers as sampled in this study (Figure 6). We estimated the ages of *Elaenia *speciation events using a mitochondrial clock rate of 2% sequence divergence/MY for reasons provided in the Discussion. The temporal reconstruction of differentiation events in *Elaenia *is depicted in Figure 6.

## Discussion

### Phylogenetic relationships within *Elaenia *and taxonomic conclusions

All analyses identified two primary clades: (1) the two savannah specialists *E. ruficeps *and *E. cristata *which received high posterior probability; and (2) the remaining species with moderate Bayesian and parsimony support (Figure 3). Within the large clade there was strong support from the concatenated data for placing *E. gigas *as sister to the rest, which contradicts vocal data and crest shape that instead suggest a close relationship with *E. flavogaster *[16].

*E. obscura*, *E. dayi *and *E. frantzii *all lack the white coronal patch present in most other *Elaenia *species, and consequently have been treated as a super-species complex by some authorities [16]. While our data corroborate the presumed close relationship between *E. obscura *and *E. dayi *[18], they strongly reject the inclusion of *E. frantzii *in this complex. The mitochondrial divergences between Andean *E. o. obscura *and Atlantic *E. o. sordida *are comparable to those between sister species of *Elaenia *(e.g. *E. spectabilis *and *E. pelzelni*; Table 3), and are ten times higher than divergences within *E. o. obscura *sampled from along a 1500 km Andean transect. Furthermore, *E. o. obscura *and *E. o. sordida *did not always form a monophyletic clade with respect to *E. dayi *(Figure 3) and are best treated as separate species: *E. obscura *and *E. sordida*.

Our analyses confirmed the genetic distinctness of *E. [chiriquensis] brachyptera *with respect to *E. [chiriquensis] albivertex *and their treatment as separate species (Rheindt et al.: Cryptic speciation in the Lesser Elaenia *Elaenia chiriquensis*, submitted). Deep genetic divisions were also observed in *E. fallax*. The level of ND2 divergence between Jamaican *E. f. fallax *and Hispaniolan *E. f. cherriei *exceeded that recorded between other species of *Elaenia*, suggesting long-term separation and species-level treatment for *E. cherriei *(Table 3; Figure 2).

*E. parvirostris *has usually been allied with *E. albiceps*, based on vocal and morphological grounds, and apparent hybridisation [16,19]. Both mitochondrial and nuclear sequences placed the two in unrelated clades; *E*. *parvirostris *in a clade consisting primarily of migratory lowland species, and *E*. *albiceps *in a clade with *E*. *pallatangae *(Figure 3). Given that *E. parvirostris *is also a lowland migratory species, its phylogenetic placement here is consistent with its ecology. Furthermore, it also shares with other members of this clade (*E. spectabilis*, *E. pelzelni*) three (as opposed to the usual two) wing-bars. The placement of *E. parvirostris *and *E. albiceps *in two relatively distant clades calls for a re-examination of the potential hybrid zone in southern Bolivia [19].

*E*. *albiceps *is also thought to hybridise with *E. pallatangae *[18]. The five recognised subspecies of *E. pallatangae *are distributed over two discrete South American land areas: the Andes and the Venezuelan Tepuis (Figure 1). All our Venezuelan samples (*E. [p.] olivina*) formed a distinct clade closely related to a number of Central American and Caribbean *Elaenia *species, while the Andean samples (*E. pallatangae sensu stricto*) emerged in a clade with *E. albiceps *and were scarcely distinguishable from the latter in both mitochondrial and nuclear DNA (Figure 3). Clearly, more detailed sampling is needed to resolve this unusual phylogenetic pattern. Future research may reveal that *E. albiceps *and *E. pallatangae sensu stricto *may be conspecific, or that genetic introgression has accounted for their similar mitochondrial DNA, and the current taxonomic status of the latter two forms is best retained until such studies have been conducted. Nevertheless, the phylogenetic divergence between *E. [p.] pallatangae *and *E. [p.] olivina *on both nuclear and mitochondrial DNA indicates treatment of *E. olivina *as a species that is not particularly closely related to Andean *E. pallatangae*.

Some reasonably deep mitochondrial divergence was found between the Cayman Island subspecies *caymanensis *of *E. martinica *and the other two subspecies investigated (Table 3). In contrast, samples of *E. martinica riisii *from Puerto Rico and *E. m. martinica *from St. Vincent were barely distinguishable from one another on ND2 divergences (Table 3). This suggests a close affinity of the latter two subspecies, which are geographically linked through the Lesser Antillean island chain. The Cayman Island race is geographically isolated, with sequence divergences typical of distinct tyrannid subspecies [17].

### Habitat shifts in the evolutionary history of *Elaenia*

Our reconstructions of ancestral habitat requirements revealed pronounced differences not only in the timing of the emergence of certain habitat preferences, but also in the frequency of habitat shifts (Figure 4). In the following, we will examine these habitat shifts individually for each habitat considered.

#### Savannah

*Elaenia *includes two habitat specialists (*E. ruficeps *and *E. cristata*) that are closely tied to the occurrence of natural savannah and cerrado. Our DNA phylogeny (Figure 3) united these two in a clade that was sister to the remaining species of *Elaenia*. Zimmer [18] also identified features of the wing formula and nostril structure that separated *E*. *ruficeps *and *E*. *cristata *from the rest of the genus. Three other species regionally breed in savannah habitat, though they are more generalist in nature and occur in other habitat types as well (Table 1). *E. flavogaster *and *E. chiriquensis *are widespread denizens of a variety of open habitats, while *E. dayi *is primarily a forest bird that also breeds in stunted tree savannah within its restricted range in the Venezuelan Tepuis [15,16]. These facultative savannah inhabitants did not form a clade and were positioned in different assemblages.

The finding that savannah specialists are sister to the rest of *Elaenia *suggests that savannah would have constituted an important part of the Neotropical landscape during the early diversification of the genus. Although parameter state reconstructions are either equivocal (MP) or argue against (BI) a savannah-inhabiting ancestor of *Elaenia*, savannah is likely to have been invaded by proto-*ruficeps/cristata *shortly after the initial divergence of *Elaenia *(Figure 4).

#### Forest interior

*Elaenia *is widely distributed throughout Neotropical edge habitats and other open vegetation and avoids tropical lowland rainforests [15,16]. Nevertheless, a number of species are found primarily or secondarily in the interior of montane or otherwise temperate forest (Table 1). Even though nine species inhabit forest, both Bayesian and parsimony state reconstructions showed that occurrence in forest interior arose on only two or three (maximally four) occasions (Figure 4). Within these assemblages of forest-inhabiting species, the prevailing pattern is one of allopatric distributions (Figure 1). This is consistent with the notion that geographical isolation rather than ecological divergence is the most common mode of speciation in Neotropical birds [1,3,6,9].

#### Riparian habitats

Almost half of the species of *Elaenia *are either narrowly confined to riparian vegetation or have a regional preference for it. Riparian habitat preferences can vary widely from montane streamside habitat (e.g. *E. strepera*) to *Cecropia *stands on Amazonian river-islands (*E. pelzelni*), but a frequent commonality of these habitats is that they constitute breaks in blocks of closed forest habitat or savannah/cerrado. Both MP and BI reconstructions indicated that an association with riparian growth may have been prevalent in the main lineage of *Elaenia *in very early stages of its diversification and may have been lost on at least two to four occasions (Figure 4). Two of these secondary losses of riparian association involve lineages that subsequently colonized forest interior where they diversified extensively (the *E. obscura/dayi *complex and the "montane forest clade" of Figure 3). River and stream association may have provided an evolutionary vehicle for lowland species of open habitat to move up the streams and colonize montane forest. This notion is further supported by the fact that the most basal riparian species *E. gigas *(Figure 3) is confined to the Andean foothills while many subsequent speciation events gave rise to lineages that occur in elevationally higher regions.

It is intriguing that although the ancestor of *Elaenia *was able to colonize montane forest, it never gained a foothold in tropical lowland forest. Brumfield and Edwards [3] suggested that competitive interspecific interactions may limit colonization potential in the evolution of Neotropical suboscines. Accordingly, *Elaenia *may have been able to exploit new montane forests formed by mountain uplift, while being excluded from the older lowland forests through competitive interactions with other flycatchers.

### Evolutionary history of migratory behavior in *Elaenia*

Some species of *Elaenia *are austral migrants that breed in the temperate zone of the southern Neotropics and move to the tropics in the austral winter (Table 1). Such migratory behavior is ordinarily displayed by the entire species (*E. spectabilis, E. parvirostris, E. strepera*), though in *E. albiceps *only the southern race *E. a. chilensis *migrates while more northerly races are sedentary. In addition, *Elaenia *contains five "partially migratory species" that are sedentary over most parts of their range but do exhibit short-distance migratory behavior in some of their more temperate populations (Table 1). MP and BI reconstructions both indicated a sedentary ancestor of *Elaenia *with migratory and partial migratory behavior arising several times (Figure 5). This result is in agreement with Joseph et al.'s [20] phylogenetic analysis of migratory behavior in *Myiarchus *flycatchers. The fact that austral long-distance migration exists in some populations of species that are otherwise sedentary, and the high incidence of partial migration in *Elaenia *species, suggest that migratory behavior is evolutionarily labile in tyrannids.

From our phylogenetic reconstructions there is one possible example of speciation resulting from loss of migratory behaviour. *E*. *pelzelni *is the only sedentary habitat specialist of a clade that comprises either migratory or partially migratory habitat generalists (Figures 3, 4). Although neither mode of reconstruction unequivocally identified an austral migratory ancestor to this clade, ecological evidence suggests that *E. pelzelni *is likely derived from an austral migrant ancestor. *E. pelzelni *has very specific habitat requirements (river-island habitat along the Amazon), and considering the migratory generalist life histories of all other members of this clade, it is very unlikely for the ancestor of *E. pelzelni *and *E. spectabilis *to have been a sedentary specialist. Though dispersing widely, wintering populations of *E. spectabilis *avoid the interior of tropical rainforest, frequenting instead marginal and riparian habitats. An ancestor with a similar behaviour could therefore have easily given rise to a sedentary riparian *E. pelzelni*.

### The timing of *Elaenia *diversification

Dating of evolutionary events has presented major difficulties in phylogeographic studies of birds. The sparse avian fossil record (especially of songbirds) complicates the assessment of the age of certain lineages. Instead, phylogeneticists have resorted to the notion of a molecular clock to date speciation events (for a summary, see [21,22]). However, the constancy and universality of a traditional avian mitochondrial molecular clock of *c*. 2% divergence/MY has been questioned [21-23], and rate constancy has been contradicted by Bayesian reconstructions using calibrations at the level of avian orders and vertebrate classes [24]. Nevertheless, a large number of avian clock calibrations provided for phylogeographic studies at the genus level seem to converge at around 2%/MY (e.g. [25-28]). More importantly, Weir and Schluter [29] used cross-validation techniques to compare 90 candidate avian clock calibrations dating back overwhelmingly to the genus level and found strong support for a 2.1%/MY clock rate constant over the last 12 MY and universal across twelve bird orders. In tyrannid flycatchers in particular, Rheindt et al. (The timing of Neotropical speciation dynamics: a reconstruction of *Myiopagis *flycatcher diversification using phylogenetic and paleogeographic data, submitted) compared mitochondrial molecular clock rates from the literature with paleogeographic calibrations of ND2 sequences within a closely related elaeniid flycatcher genus (*Myiopagis*), and found that the traditional avian molecular clock of 2% mtDNA divergence/MY is a reasonable assumption for elaeniid ND2 sequences. Based on these considerations, we have used a mitochondrial clock rate of 2%/MY to date speciation events in *Elaenia *(Figure 6). We emphasize that these dating estimates can only be viewed as an approximation, and we only discuss them in the rough timeframe of earth-historical epochs.

According to our reconstructions, the earliest divergences occurred in rapid succession in the late Miocene (c. 8.5 to 5.3 MYA). Arising during this period were the two savannah specialists as well as *E. gigas*, *E. strepera*, the *E. obscura/dayi *complex, the migratory lowland clade (Figure 2), and the lineage comprising all the remaining species. The Amazon lowlands at that time were intermittently flooded by "Lago Amazonas", an extensive spatially and temporally variable freshwater lake system that drained into the Caribbean (see [30] and references therein). The landscape constituted "...a vast complex of shallow mega-lakes surrounded by swampy grassland savanna..." interspersed with forest (p. 206 in [30]). Such a dynamic savannah-forest landscape with water barriers rapidly changing in extent was likely conducive for vicariant speciation events such as those that led to the two savannah specialists and the migratory lowland lineage (Figure 3). The Andes at that time were only about a third to half of their current elevation [31,32]. Consequently, the montane *Elaenia *lineages that had emerged by that time were either Andean foothill species (e.g. *E. gigas*) as opposed to Andean highland species, or were clades from ancient Neotropical mountain shields such as *E. dayi *from the Tepuis or *E. sordida *from the Brazilian Shield, which may have given rise to Andean *E. obscura *at a much later date.

With the onset of the Pliocene at c. 5 MYA, there was a second wave of speciation events which commenced with the differentiation of the lowland migratory clade (Figure 3) into its three main lineages, two of which include austral breeders that annually migrate into the tropics (*E. parvirostris *and *E. spectabilis*). This and a similar lowland speciation event between *E. mesoleuca *and the *E. chiriquensis *complex fell within a period of global warming [33-39] and concomitant sea level rises of up to 80 m [40] which led to pronounced marine incursions into the Río de la Plata Basin in the south and the Amazon in the north. Such marine incursions or the continuing spatio-temporal dynamics of Lago Amazonas further inland, or a combination thereof, were presumably the driving forces for these lowland speciation events.

The mid to late Pliocene from c. 4 MYA onwards sees the emergence and rapid differentiation of the "montane forest clade" (Figure 3), giving rise to a suite of highland species. This coincides with a period of rapid Andean uplift between 5 and 2 MYA [31]. The Central American mountains were colonized by *E. frantzii *immediately after the closure of the Panamanian isthmus at c. 3.5 MYA [41,42]. Starting around the same time, the Caribbean was invaded by *E. fallax, E. cherriei *and *E. martinica*, though we are unable to specify whether one or two colonization events were involved. Dispersal must certainly have played a role in the diversification of the montane clade, as neither the Caribbean islands nor the Venezuelan Tepuis (colonized by *E. olivina*) have ever been connected to the Andes. The Andes likely played a role in isolating the Chocó endemic *E. brachyptera *from cis-Andean *E. chiriquensis *around the late Pliocene.

## Conclusion

Our ancestral reconstructions of habitat preferences in *Elaenia *reveal pronounced differences not only in the timing of the emergence of certain habitat preferences, but also in the frequency of habitat shifts. Savannah specialism has an early origin in the genus, which highlights the importance of this habitat in Neotropical Pliocene and late Miocene biogeography. Forest in old mountain ranges such as the Tepuis and the Brazilian Shield was colonized early on with limited subsequent in-situ differentiation, while the most important colonization event of montane forest was in conjunction with Pliocene Andean uplift and led to a rapid allopatric diversification. Riparian habitats may have played an important role in facilitating the colonization of new habitats by enabling birds to expand up the mountains along streams and to adapt to newly emerging montane forest habitat. We also confirm austral long-distance migration to be a labile trait in Neotropical songbirds.

## Methods

### Sampling regime and laboratory techniques

We investigated one mitochondrial coding gene region, NADH dehydrogenase subunit 2 (ND2), and one nuclear intron, β-fibrinogen intron 5 (Fib5). Tissue samples were obtained for all but one currently recognized species of *Elaenia*, as *E. ridleyana*, endemic to the tiny island of Fernando de Noronha in the Atlantic Ocean, was not available for inclusion. Sampling focussed on obtaining a large geographic and subspecific coverage of *Elaenia *species. In addition to the 64 specimens sampled in the present study, sequence data were sourced from the literature for a further nine samples (see Table 2), thereby totalling 18 species. Specimen information and Genbank accession numbers are provided in Table 2. For outgroup comparisons we used *Capsiempis flaveola *[43] (Genbank accession numbers DQ294563 and DQ294475), as it has a close affinity to *Elaenia *[17].

Extraction and sequencing techniques followed Rheindt et al. [17]. ND2 sequences were obtained for all 73 ingroup samples, while the Fib5 dataset was restricted to 47 samples. Alignment and editing of sequences was carried out using SEQUENCHER v.4.1.4 (Gene Codes Corp., Ann Arbor, Michigan). Coding sequences were translated and checked for anomalous substitution patterns, such as double peaks and stop codons. All sequences were manually edited and assessed for deviant base composition.

### Phylogenetic analysis

Phylogenetic analysis of the separate data partitions as well as the concatenated dataset was carried out using maximum parsimony (MP) and Bayesian inference (BI). Indels were excised from the dataset on account of their low number and short lengths, but the parsimony-informative ones were later mapped onto the tree. For MP analysis, we ran heuristic searches (default settings activated unless otherwise specified) using the program PAUP* v4.0b10 [44]. Tree searches were unweighted as there was no evidence of saturation (see Results). PAUP* was also employed for estimating partition and sequence parameters. All PAUP* analyses involved 100 bootstrap replicates.

For BI analysis, the hierarchical likelihood ratio test implemented in the program MODELTEST 3.06 [45] was run to recover an appropriate evolutionary model for each partition (Table 4). MRBAYES 3.1 [46] was run with default settings (unless otherwise specified) using the basic parameters determined by MODELTEST, while allowing for the estimation of model-specific parameters such as base frequencies or gamma shape [p. 56 in ref [47]]. The analysis of the concatenated dataset was conducted with separate evolutionary model settings for each partition. BI searches employed Metropolis-coupled Markov chain Monte Carlo sampling with one cold and three heated chains running for 1 million generations with a sampling frequency of 100. The burn-in period was determined graphically following Ronquist et al. [47] and samples within this period were discarded (Table 4). Subsequently, posterior probabilities were derived from the 50% majority rule consensus of all trees retained.

### Ancestral character state reconstruction

Ecological parameters were mapped onto the tree topology derived from the concatenated dataset (with species-level lineages reduced to one representative) as discrete characters using life-history information from Ridgely and Tudor [15] and Hosner [16]. Transition rates between individual parameter states were also calculated. Four ecological parameters were mapped. (a) Breeding occurrence in forest interior (yes/no). Species that mainly breed in forest edge were not included; (b) Breeding occurrence in natural savannahs and cerrado vegetation (yes/no). Species ranging in similar open habitat of anthropogenic origin were not included. (c) At least regional breeding occurrence in riparian habitats such as gallery forest, Amazonian river-islands, riparian successional zones, Andean and pre-Andean streamside and similar habitats (yes/no). (d) Migratory status (austral long-distance migrants, partial/short-distance migrants or sedentary species). This character was frequently double-coded, since all species that have partially migratory populations are sedentary throughout most of their range, and one species (*E. albiceps*) includes both sedentary montane populations and austral long-distance migrants.

For state reconstruction of these parameters, we employed an MP-based approach [48] as implemented in the program MACCLADE[49]. MP analysis assumed unordered states. Character states were also mapped using the program MULTISTATE in the computer package BAYESTRAITS[14]. The Bayesian method generates posterior probabilities for ancestral character states to gauge estimation reliability. BI analyses were run for 1,000,000 iterations with a tree sampling rate of 1,000 and a burn-in of 50,000 iterations. We employed a uniform hyperprior to obtain a suitable prior for Bayesian runs assuming an exponential distribution. Posterior probabilities for character states were recorded for relevant nodes, and character transition rates were estimated.

### Molecular clock estimates

PAUP* was used in conjunction with the evolutionary model specified by MODELTEST 3.06 for our ND2 partition (Table 4) to compute maximum-likelihood scores for the most likely tree under the enforcement of a molecular clock and under relaxed branch length assumptions. For computational efficiency the ND2 dataset was reduced to one representative per species-level lineage. Likelihood scores were compared with a χ^2^-test (df = 20) to see whether they differed significantly. We refrained from using the concatenated dataset for this analysis, because the molecular clock rate used in this study (2% divergence/MY) is based on mitochondrial DNA (see Discussion for rationale).

## Authors' contributions

FER designed the study, carried out labwork and phylogenetic analyses and drafted the manuscript. LC assisted with the design of the study and contributed to the manuscript. JAN assisted with the design of the study and labwork and contributed to the manuscript. All authors read and approved the manuscript.
